# Editorial: The complement system in autoimmunity

**DOI:** 10.3389/fimmu.2022.1085525

**Published:** 2022-11-16

**Authors:** Eveline Y. Wu, Jessy J. Alexander, Shoichi Fukui

**Affiliations:** ^1^ Division of Rheumatology, Department of Pediatrics, University of North Carolina, Chapel Hill, NC, United States; ^2^ Division of Allergy/Immunology, Department of Pediatrics, University of North Carolina, Chapel Hill, NC, United States; ^3^ Department of Medicine, University of Buffalo, Buffalo, NY, United States; ^4^ Program in Cellular and Molecular Medicine, Boston Children’s Hospital, Harvard Medical School, Boston, MA, United States; ^5^ Department of Pediatrics, Harvard Medical School, Boston, MA, United States; ^6^ Department of Immunology and Rheumatology, Nagasaki University Graduate School of Biomedical Sciences, Nagasaki, Japan; ^7^ Department of General Medicine, Nagasaki University Graduate School of Biomedical Sciences, Nagasaki, Japan

**Keywords:** complement - immunological terms, complement activation, alternative complement pathway, autoimmunity, complement therapeutics

Complement is a major effector mechanism of the innate immune system and also augments adaptive immune responses. The complement system comprises more than 30 proteins in plasma and on cell surfaces and can be activated *via* the classical, alternative, and lectin pathways. This article collection reviewed important contributions and highlighted novel roles of complement and its activation in various immune-mediated conditions. Several major themes emerged including: (i) levels of complement may predict disease course, suggesting complement may also serve as a therapeutic target, (ii) polymorphisms in complement genes may influence disease risk, and (iii) complement may have a pathogenic role in unexpected immune-mediated conditions like interstitial cystitis and Leydig cell senescence that warrants further study.

Traditionally, complement activation was known to occur extracellularly and defend against pathogens systemically. With increase in our understanding, it has become evident that complement activation also occurs intracellularly, affecting every organ from brain to the urinary bladder. The complement system and inflammasomes link both adaptive and innate immune systems and are involved in autoimmunity (1–5). Complement stimulates secretion of IL-1β that is often elevated in autoimmunity (6) *via* the NLRP3 inflammasome. Studies by Kiran et al. suggest that inflammasome inhibition alleviates disease pathology by suppressing adaptive immune cells in interstitial cystitis, a condition with significantly depleted C4 levels (7) and associated with autoimmune disease.

Li et al. also showed a potential surprising contribution of complement activation in Leydig cell senescence during an inflammatory state. Li et al. demonstrated that Leydig cell senescence occurred in the chronic inflammatory environment of a murine model of experimental autoimmune orchitis (EAO). Leydig cell senescence, in turn, antagonized androgen synthesis. Leydig cells were analyzed by high throughput scRNA-seq, and complement proteins and membrane-bound complement regulatory proteins were found to be prominently expressed in testicular Leydig cells undergoing senescence and negatively correlated with androgen synthesis.

Fully understanding the role of the complement system in immune-mediated disorders may provide insight into complement as a therapeutic target in the future. Kimoto et al. provided a nice review of the contribution of the alternative complement pathway in the pathogenesis of anti-neutrophil cytoplasmic antibody (ANCA)-associated vasculitis (AAV) and therapeutic perspectives on AAV from basic to clinical studies. They also summarized clinical trials on avacopan (CCX168), a C5a receptor (C5aR) antagonist, to control the disease and reduce the side effects of corticosteroids. Their review helps readers understand a success story of how the contribution of the alternative complement pathway in AAV was unveiled from mouse models to humans, leading to the development of a novel therapeutic option as a great example of a so-called “from bench to bedside” approach.

Fujita et al. demonstrated a relationship between the existence of hypocomplementemia at diagnosis of immunoglobulin G (IgG) 4-related disease (IgG4-RD), clinical manifestations, and laboratory data. Furthermore, the authors revealed a more frequent relapse after remission of the disease in patients with hypocomplementemia. This study suggests the difference in the pathogenesis of IgG4-RD between patients with and without hypocomplementemia. Considering elevated levels of C5a in IgG4-RD by a previous report in conjunction with this study (8), IgG4-RD might be a candidate disease in which a medication targeting complement, including avacopan, is expected to be beneficial.

Another condition where complement has recently been implicated and may serve as a therapeutic target is multiple sclerosis (MS) (9). In this issue, Linzey et al. showed that in MS, the disease pathology is modulated by the classical pathway in the progressive model, and by the alternative pathway in the relapsing encephalomyelitis model. It is critical to gain a comprehensive insight into the contributions of the complement system in MS to define effective therapies in each setting.

Immune complex deposition and activation of complement via the classical pathway are well-known contributors in immune-complex proliferative glomerulonephritis (GN) (10). Alternative complement pathway activation may also augment tissue injury in GN (11). Gouda et al. performed targeted next-generation DNA sequencing on genes encoding proteins involved in complement regulation including complement factor H (CFH) and membrane cofactor protein (MCP). They identified genetic variants in *CFH* and *MCP* genes in Egyptian patients with immune-complex proliferative glomerulonephritis (GN) including lupus nephritis and post-infectious GN (PIGN). Further study is needed to determine the functional and clinical implications of these variants.

Activation of the alternative complement pathway is also thought to have an important role in IgA nephropathy (IgAN) (12). Given that complement factor B (CFB) is an initial factor in alternative complement pathway activation, Shi et al. studied whether variants in the *CFB* gene confer susceptibility to IgAN in Han Chinese. They identified a *CFB* genetic variant associated with less alternative complement pathway activation in IgAN and conferred low risk of IgAN.

Lastly, we would like to thank the reviewers for their time and valuable assessments. This important collection of articles on the Research Topic “*The Complement System in Autoimmunity*” would not be possible without their contributions.

## Author contributions

EW, JA, SF planned, wrote, and revised the editorial manuscript. All authors contributed to the article and approved the submitted version.

## Funding

EW work was supported by the National Center for Advancing Translational Sciences, National Institutes of Health, through Grant KL2TR002490. The content is solely the responsibility of the authors and does not necessarily represent the official views of the NIH.

## Conflict of interest

The authors declare that the research was conducted in the absence of any commercial or financial relationships that could be construed as a potential conflict of interest.

## Publisher’s note

All claims expressed in this article are solely those of the authors and do not necessarily represent those of their affiliated organizations, or those of the publisher, the editors and the reviewers. Any product that may be evaluated in this article, or claim that may be made by its manufacturer, is not guaranteed or endorsed by the publisher.
